# Malignant Schwannoma of Anterior Abdominal Wall: Report of a Case

**DOI:** 10.4021/jocmr2009.09.1264

**Published:** 2009-10-16

**Authors:** Zhamak Khorgami, Shirzad Nasiri, Freshteh Rezakhanlu, Nassim Sodagari

**Affiliations:** a General surgery, Tehran University of Medical Science, Tehran, Iran

## Abstract

Malignant schwannoma of the anterior abdominal wall nerves is extremely rare. Malignant peripheral nerve sheath tumors (MPNST) represent approximately 10% of all soft tissue sarcomas and it is found in 4% of patients with neurofibromatosis 1. We present a case of malignant schwannoma in a 28-year-old female patient with neurofibromatosis 1. She presented with a painful mass in the right upper quadrant of her abdomen. The tumor location was in the abdominal wall in explorative laparatomy and malignant schwannoma was diagnosed in pathologic assessment. The tumor recurred in 3 months and computed tomography showed two masses in the right side of abdominopelvic cavity. Thereafter, second complete surgical resection was performed and pathologic finding was the same. In spite of administering chemotherapy after second surgery,the tumor recurred and magnetic resonance imaging finding showed a huge heterogeneously enhancing mass with adhesion to the inner side of the abdominal wall. The patient died because of acute respiratory failure due to multiple bilateral pulmonary metastases. Tumor location and rapid recurrence was unique in our patient.

**Keywords:**

Malignant peripheral nerve sheath tumor; Malignant schwannoma; Abdominal wall

## Introduction

A 28 years old woman with recurrent malignant schwannoma originating from anterior abdominal wall nervesis reported in our article. Malignant peripheral nerve sheath tumor also known as malignant schwannoma, are highly malignant sarcomas that tend to arise in the head and neck region or on the exterimities, and only very rarely in anterior abdomional wall. Almost 50% of cases are associated with neurofibromatosis. Although various radiologic imaging methods are helpful for identifying some features of the mass, definitive diagnosis requires histological examination and immunohistochemical staining. After treatment, the tumor recurs in 25% of patients. Five-year survival rates as high as 80% have been reported. Total excision and lack of invasion of surrounding tissue and vessels and absence of neurofibromatosis are features associated with better outcome

## Case presentation

**Figure 1:**
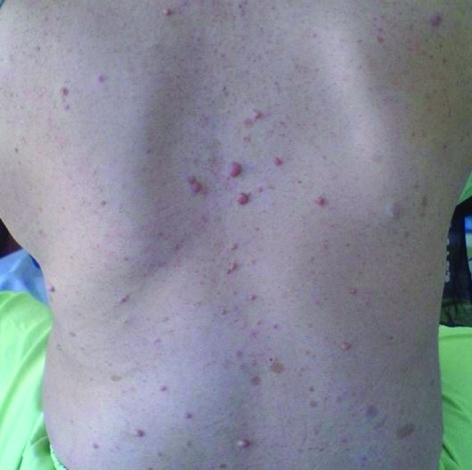
Multiple cafe-au-lait spots scattered over the patient's entire body.

A 28 years old female was admitted for management of increasing abdominal distention and sustained pain in right upper quadrant of abdomen for two months. Her medical history included type 1 neurofibromatosis (NF1), also known as von Recklinghausen disease. She had not history of surgery or radiotherapy. On physical examination, there were multiple cafe-au-lait spots scattered over the patients entire body and axillary freckling, but no cutaneous neurofibromas was present (Fig. 1). Lisch nodules were not detected in the iris by a slit lamp. A large firm fixed mass was palpated in the right portion of the abdomen.

**Figure 2:**
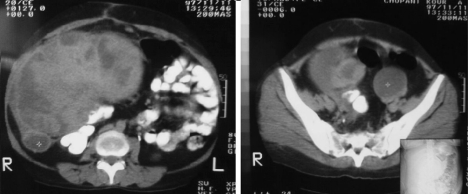
Abdominopelvic Computed Tumography shows a large mass in the right side of abdominal cavity.

The sonographic and computed tomography findings showed a heterogenic solid mass in right upper quadrant (RUQ) of abdomen that extended down to the right lower quadrant (RLQ) of abdomen (Fig. 2). Laboratory data was in normal range. Thereafter the patient underwent a surgical resection. During the operation, an encapsulated, white grayish, bulky, fish fleshy mass attached to the abdominal wall measuring 16 x 13 x 6 cm was detected in the right side of abdomen. There was no local invasion, and the tumor was resected en block. The cut surface showed cystic and solid components with hemorrhagic foci. Pathologic findings showed atypical spindle cells with mild pleomorphism and high mitotic activity that suggest malignant peripheral nerve sheet tumor (malignant schwannoma). Patient did not follow the recommendations for postoperative adjuvant therapy. After three months, she came with local recurrence of tumor.

**Figure 3:**
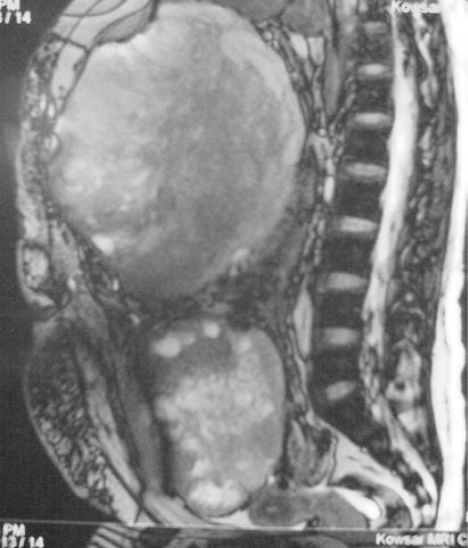
Magnetic Resonance Imaging shows huge heterogeneously enhancing mass with some necrotic foci which has filled the abdominal and pelvic cavities.

Radiologic findings showed two masses in the right anterior pararenal space extending down to the lower abdomen. Thereafter the patient underwent second surgical resection. During the second operation, one white grayish well limited fish fleshy tumor measuring 6.1 x 5.5 x 4.7 having whitish out surface with gelatinous foci and the other mass measuring 2.1 x 1.9 x 1.8 cm, were detected in the abdomen and resected. Section showed a high grade spindle cell hypercellular sarcoma composed of rather monotonous spindle cells having atypical hyperchromatic nuclei with foci of perivascular palisading. Histological examination of the resected specimen revealed the tumor to be malignant schwannoma. The patient received chemotherapy (cyclophosphamide) for 2 months but there was tumor recurrence in less than two months. Magnetic resonance imaging showed the recurrent tumor as a huge heterogeneously enhancing mass with some necrotic foci which has filled the abdomen and pelvic cavities. The mass was located in right side of the abdomen with midline extension (Fig. 3).

There was direct contact with liver and gallbladder which were pushed superiorly. Adhesion to the inner side of the abdominal wall was seen. Retroperitoneal extension and lymphadenopathy was not noted. As a result, the tumor was considered to arise from the anterior abdominal wall nerves. The patient died about two months later because of respiratory failure due to widespread bilateral pulmonary metastasis.

## Discussion

The occurrence of intraabdominal and retroperitoneal schwannomas is extremely rare. Intraabdominal schwannomas occur most frequently in the alimentary tract [1]. Gastrointestinal involvement occurs in about 10% to 25% of patients with NF1 and includes solitary or multiple neurofibromas, leiomyomas, and rarely, plexiform neurofibromas [2].

Neurofibromas are tumors derived from Schwann cells, fibroblasts, and supporting cells known as perineural cells. Typically, theyare benign and manifest as multiple tumors. NF1 is an autosomal dominant genetic disorder with a prevalence of approximately 1 in 4,000 births and no racial predilection. NF1 is characterized by multiple neurofibromas along the peripheral nerves, optic nerve gliomas, sphenoid wing dysplasia, pigmented lisch nodules,and hyperpigmented macular skin lesions known as cafe-au-laitspots. It is associated with a gene on chromosome 17. Theformation of dermal neurofibromas is a hallmark of NF1 witha characteristic distribution on the trunk and sparing of theextremities. With time, neurofibromas may undergo malignantdegeneration [3].

At gross examination, neurofibromas appear as firm, gray-whitemasses. At histologic analysis, neurofibromas contain Schwanncells and nerve fibers that grow in a disorganized fashion andappear as interlacing bundles of elongated cells with intracellular collagen strands.

There are three types of neurofibromas: localized, diffuse, and plexiform. The vast majorities of these lesions are localized and have no association with NF1, althoughany of these lesions may occur in NF1 [4]. Localized neurofibromas are well-delineated, firm lesions with a white and shiny surface and may appear in the dermis or subcutaneous tissues. Plexiform neurofibromas are thick, fusiform, ropelike expansions of nerve roots and peripheral nerve fibers with a mucoid or translucent cut surface. They are generally unencapsulated tumors that blendindistinctly with adjacent connective tissue or peripheral nerves and are diagnostic of NF1. Their gross appearance is often described as a "bag of worms". Rapid enlargement of an existing neurofibroma may be a sign of malignant transformation until proved otherwise[3]. A malignant peripheral nerve sheath tumor (MPNST) is now the preferred name for the spindle cell malignancy of peripheral nerve schwanncells. Malignant schwannoma are the malignant variants of schwannomas and neurofibromas [5]. MPNSTs most commonly occur in the deep soft tissues, usuallyclose to a nerve trunk. The most common sites are the sciaticnerves, brachial plexus, and sacral plexus. The past literature referred to MPNST as malignant schwannoma, neurogenic sarcoma, and neurofibrosarcoma. MPNST is the current term used by the World Health Organization for this highly aggressive tumor. MPNSTs may arise from plexiform neurofibromas, de novo or secondary to radiation therapy. At histologic analysis, the presence of mitotic figures distinguishes MPNST from otherwise typical neurofibromas [2]. It represents approximately 10% of all soft tissue sarcomas and its diagnosis has been called one of the most difficult and elusive diagnoses in soft tissue diseases. It is found in at least 4% of patients with neurofibromatosis 1, where its developments are to be a multi-step, multi-gene process.

Conversely, up to half of all cases of MPNST are diagnosed in persons with neurofibromatosis 1. About one in ten cases are associated with irradiation [6].Tumor is usually found in the lower extremities, but one-ninth of all lesions occur in the head and neck region, usually associated with the large cranial nerves. MPNSTs occur rarely in the retroperitoneum [5]. Lesions related to neurofibromatosis typically occur a decade or more earlier than those in non-syndrome patients. MPNST occurs usually in 20-50 years of age, but children and elderly may also be affected. Clinically, pain is a classic presenting symptom in MPNST. Other findings include masses larger than 2 - 6 cm with irregular borders and a history of rapid growth. Often, MPNST produces neurologic deficits in the distribution of theinvolved nerves due to impingement or mass effect. Atcomputed tomography, the attenuation of neurogenic tumors depends on their histologic characteristics. Neurofibromas typically have low attenuation related to the fat content of myelin from Schwann cells, the high water content of myxoid tissue, entrapment of fat and cystic areas of hemorrhage and necrosis. Central enhancement or a target appearance may be seen due to the less cellular and vascular myxoid tissue located in the periphery and more vascular fibrous tissue seen centrally. The characteristicdumbbell lesion, a partly intradural and partly extradural tumor, represents a neurofibroma that expands the intervertebral foramina and may be best appreciated with cross-sectional imaging. Ultrasound of a neurofibroma reveals a hypoechoic, well-defined mass [3]. Although various radiologic imaging methods are helpful for identifying some features of neurofibroma, definitive diagnosis requires histologic examination and immunohistochemical staining [5]. Radiologically, MPNSTs and neurofibromas may appear indistinguishable; however, certain modalities are providing insight for differentiation. Gallium-67 citrate imaging has shown that MPNSTs have greater uptake compared with benign lesions. At magnetic resonance imaging,the different signal intensity characteristics of lesions witha higher degree of anaplasia are proving useful as well. Otherfactors such as a more rapid and infiltrative growth patternare particularly helpful in differentiating these lesions [7].

Surgical resection is the first line of therapy, ideally withtotal removal of the tumor. Gross inspection of MPNSTs reveals a fusiform, fleshy, tan-white mass with areas of degeneration and secondary hemorrhage. The nerve proximal and distal to the tumor may be thickened due to spread of the tumor along the epineurium and perineurium. At histologic analysis, MPNSTs are unencapsulated infiltratingtumors composed of spindle cells arranged in a whorling pattern with irregular nuclei, cyst formation, and nuclear palisading. Mitotic figures are readily visible, with more than one per high-power field, and 50% - 90% of cases are immunoreactive with S100 protein staining. Owing to a high risk of recurrence with incomplete resection, postoperative irradiation and chemotherapy are necessary; however, they are often used as adjuvant therapy even if the tumor is completely resected. Even with aggressive therapy, local recurrence of tumor is seen in 50% of patients. Hematogenous metastatic spread occurs most commonly to the lungs. The reported 5-year survival rate for patients withMPNST without NF1 is as high as 50%. It drops to as low as 10% for MPNST patients with NF1 [2]. The five-year survival rate of malignant schwannoma is low, primarily due to poor response of the tumors to available treatments and metastasis to the lungs and other organs. Patients that usually have the best outlook are those that are young and have relatively small tumors able to be completely removed via surgical means. Total excision, lack of invasion of surrounding tissues and vessels, and absence of neurofibromatosis, are features associated with better outcome [5]. Genetic counseling of family members suspected to have thisdisease should be performed [3].
